# Overlooked and unaddressed: A narrative review of mental health consequences of child marriages

**DOI:** 10.1371/journal.pgph.0000131

**Published:** 2022-01-12

**Authors:** Rochelle A. Burgess, Mairi Jeffery, Sabina Adhiambo Odero, Kelly Rose-Clarke, Delanjathan Devakumar

**Affiliations:** 1 Institute for Global Health, University College London, London, United Kingdom; 2 Department of Global and Social Medicine, Kings College London, London, United Kingdom; Michigan State University, UNITED STATES

## Abstract

Child Marriage (before the age of 18) affects over 12 million young women globally, annually. Despite acknowledgement of the negative impacts of the practice on reproductive health, mental health consequences are largely overlooked. Given the ability for poor mental health to intensify other health and social challenges, understanding the mental health consequences linked to child marriage is vital. Our study is the first to examine how mental health is approached in current literature on child marriage. Our conceptual framework was informed by a rapid assessment of key issues in the field. Systematic searches of papers published between 2000–2020 were completed on four electronic databases with no language restrictions. Our protocol was registered on Prospero (CRD42019139685). Articles were assessed using PRISMA guidelines, and their quality assessed using the Joanna Briggs Institute Critical Appraisal Tools. Of the 4,457 records identified, 21 papers meeting inclusion criteria were analysed using narrative synthesis. The final sample included 5 qualitative, 1 mixed-methods and 15 quantitative studies (14 cross-sectional and 1 longitudinal study) reporting on data from 12 countries, largely in the global south. Intimate partner violence, poverty, challenges in childbirth and isolation were identified as social factors linked to emotional distress by those married as children. Depression was the most reported mental disorder. Anxiety, phobias, psychological distress, substance misuse, negative well-being and anti-social personality disorder were reported less frequently. Findings highlight that while significant emotional distress and specific mental health conditions are linked to child marriage, gaps in our understanding remain. Future studies are needed to; clarify directionality in these relationships; understand the mental health needs of young men, LGBTQI communities and those in humanitarian settings. Given the well documented cyclical relationship between social determinants and mental health conditions, we outline a series of community-oriented interventions which blend psychological, social and structural support to promote mental health and wellbeing in the contexts of child marriage.

## 1. Introduction

Child or early marriage–defined as marriage under the age of 18 –is a harmful practice that occurs globally and can limit the developmental outcomes of girls, and children born into these unions [1]. Current estimates suggest that one in five girls are married before the age of 18, and more than 650 million adult women alive today were married as children [2]. South Asia and sub-Saharan Africa bear the largest burdens of child marriages. In South Asia, where close to 1 in 5 are married before 18 [3], a recent drop in prevalence from 50% to 30% has been attributed to strategies in India, such as improvements in girls’ education and standards of living [4]. In Sub-Saharan Africa, 40% of women are married as children [2], and 18 of the top 20 countries with the highest prevalence are found in the continent. However, prevalence varies quite widely, from 2% in Tunisia to a high of 76% in Niger [3]. Though most prevalent in low income regions, Child Marriage (CM) also occurs in high income settings, such as Canada and the US where prevalence is higher among rural and minority populations who are more exposed to poverty and social inequalities [5].

Tackling CM is a global priority [6]. Sustainable Development Goal 5 (SDG5) requires bringing an end to all harmful gendered practices, including early and forced marriage, by 2030 [7]. Such efforts will require addressing other social challenges linked to the practice, for example poverty, lack of education [8], and domestic and sexual violence [9]. Interests in the health impacts of the practice have been dominated by an interest in sexual and reproductive health outcomes. For example, a 2019 global narrative review identified five broad categories of risk in this area: vulnerability to Sexually transmitted infection; cervical cancer; family planning, physical and sexual violence. In South Asia specifically, marriage before 19 has been associated with frequent childbearing, unwanted pregnancy, low uptake of contraception and maternal health services [10]. A study of 97 countries highlighted that CM posed significant risk for HIV, infant mortality and maternal health given higher rates of unprotected sex among young wives who have difficulties negotiating condom use when husbands have multiple partners, and refusal of unwanted sex [11]. A more recent analysis of teenage childbearing and HIV in Sub-Saharan Africa found that across the region, teen marriage was significantly associated with fewer years of education, early childbearing, and lower rates of HIV testing and awareness [12].

Comparatively little attention has been given to the mental health needs of those married in childhood. Seminal work by Raj and Boehmer [11] alludes to depression and suicide in their model of vulnerabilities linked to females in CM, and others have recently called for more evidence in this area [13]. Yet, significant gaps remain in understanding morbidity, experiences of distress, and the direct and indirect consequences of CM on mental wellbeing. It is at this juncture that our paper makes its contribution. We present a narrative review of literature published on the mental health CM relationship in the past 20 years, with the aim of exploring the following research question: *What are the mental health impacts (including emotional distress) associated to child and early marriage described in literature*?

### 1.1 What we do know: The socio-structural realities of child marriage

Much work has explored the complex social dynamics contributing to CM, highlighting legislative, community and household dynamics (norms) and structural and political factors. For example, the Sexual and Reproductive Health Trust Africa (SAT) global review of laws on age of consent found that the age of consent ranged from 14–21 years [14]. In Malawi for example, where 42% of girls are married early, punitive legislation for intercourse before the age of consent applies only to girls. As a result, in many countries, marriage emerges as a pathway to enable or validate sex before the age of consent.

Poverty is widely cited as a key driver of CM. Nour [15] positions it as one of three key drivers globally, alongside protection of young girls and reinforcing social ties. A recent study exploring the relationship between poverty, education and CM in India, highlighted inverse relationships between CM and household income [16], findings which have been seen elsewhere, including high-income settings [5]. Recent work from the International Centre for Research on Women (ICRW) in Kenya, Senegal, Uganda and Zambia highlighted that lack of economic opportunities contributed to higher school drop rates, which increases risk for early marriage [17]. A UNICEF report found a strong association between CM and school early drop-outs in South Asia [18]. In India, CM is a route to financial security for girls with limited employment opportunities [19].

Power relationships and agency are also linked to CM contexts and consequences. Agency is an increasingly popular concept within global health spaces, and is defined broadly, as the ability for actors to make choices, actions and decisions on the direction of their lives and livelihoods, facilitated by the presence and deployment of resources to achieve desired ends [20]. Economic analysis from Nepal and Niger suggests that CM did not independently significantly impact a woman’s future earnings, but this was mediated by the effect of CM on educational attainment [21]. Raj [22] perspectives on reduced agency in early marriages notes that large age gaps contribute to a reduced ability to negotiate the terms of sex, number or spacing of children.

Furthermore, girls are often expected to prove their fertility through pregnancy immediately after marriage and frequently afterwards [22]. Power dynamics influencing CM extend beyond family and household relations, as geopolitical realities such as conflict shape the practice globally. For example, of the top ten countries with the highest CM rates, nine are ranked as fragile states [23]. In such contexts, growing evidence suggests that girls face higher risks of CM via abduction, rape and forced marriage by terrorist groups. In Somalia for example, many parents were threatened or killed if they objected [2, 24].

### 1.2 Mental health: The missing piece?

In order to fully understand the mental health and CM relationship, we must also consider how related social environments such as those described above establish mental health risks in their own right, potentially establishing compounded risk profiles for those who experience CM.

For example, increasing evidence suggests that poverty is significantly associated to poorer mental health outcomes, particularly in relation to common mental disorders such as depression and anxiety [25–28]. The relationship between mental health, intimate partner violence (IPV) and gender-based violence is widely acknowledged [29]. A 2018 systematic review and meta-analysis of cohort studies found that IPV is associated with increased rates of depression among adolescents and young women [30]. Similar findings have been seen in South Africa [27, 31] and Uganda [32] where 1 in 10 girls are married before 15 [2].

Sexual health issues such as HIV [33] and early child-bearing [34] have clear links to poor mental health. Obstetric fistula, one of the leading reproductive health risks facing girls who marry and have children early [15, 35], has also been linked to poor mental health in numerous studies [29, 36–38]. The importance of schooling and social connectedness for adolescent mental health and wellbeing is also globally recognised [37, 39]. Such evidence suggests that the wider contexts of CM may establish particular risk pathways for poor mental health and leads us to our current work.

### 1.3 Theoretical framework

Understanding relationships between mental health and CM requires an understanding of psychiatric and social factors. Thus, our approach to identifying potential mental health consequences was guided by McGorry and van Os [40] staging approach. Through shifting the focus from diagnostic specificity towards temporal processes of symptom development, it enables space for practitioners to acknowledge and respond to general distress often linked to social determinants that often precede mental health disorders, which many argue is key to early intervention efforts [40]. In an expansion of this model, Patel and colleagues [41] describe four stages (see Table 1) which allows for assessment of evidence related to specific mental health conditions (stages 2–4) and general emotional distress (stages 0–1) linked to CM contexts.

**Table 1:** Staged approach to classification and treatment of mental health disorders.

Stage	Classifications	Treatment
0–1	Asymptomatic and non-specific mental distress	Public health strategies, including self-help awareness
2–4	Fully defined syndromes at various severity levels	Specific mental health interventions across range of health settings

*Note*: Adapted from Patel et al., [41].

A systematic review of grey literature was not included in this review, as our aim was to explore the state of current literature most likely to shape intervention design in mental health spaces, which consistently prioritises peer reviewed evidence. However, we conducted a rapid assessment of grey literature and commentaries published on the wider health consequences of CM to identify potential health factors that could drive distress related to the practice. The rapid assessment (conducted by SAO and RAB in April 2019) identified key works by Raj and Boehmer [11] and Svanemyr et al. [13] along with reports from leading NGOs in the field. This assessment identified broad priority areas in the CM field including: reproductive health needs, HIV/AIDS, Intimate Partner Violence (IPV), obstetric fistulae, social isolation and self-immolation [13, 41] which guided our search strategy.

## 2. Methods

### 2.1 Identification of relevant studies

A literature review was completed using systematic methods with no language or publication restrictions by SAO between July and August 2019 using PubMed, Embase, PsycINFO, Scopus and Web of Science databases. All articles published after 2000 were included to reflect the wider shifts in discourse which prioritised mental health created by the launch of the 2001 World Health Report [42]. In order to verify the findings from the first assessment of materials, MJ repeated the review, using the same search terms and parameters in February 2020. This identified an additional 7 papers meeting inclusion criteria published following the initial search. The protocol was registered on PROSPERO (CRD42019139685).

We used Boolean operators to explore terms related to the following three concepts informed by the scoping review: (1) child (2) marriage and (3) mental health. Similar words and phrases for child marriage (such as “early marriage”, “prepubescent female marriage”, “child bride”, “teenage marriage” and “adolescent marriage”) were included. A sample of full search terms is described in S1 Appendix.

### 2.2 Drivers of emotional distress

A search for the following associated concepts identified from the rapid scoping of literature included: intimate partner violence, sexual violence, self-immolation, obstetric fistula, and HIV/AIDS.

### 2.3 Selection of relevant studies

#### 2.3.1 Inclusion and exclusion criteria

Table 2 details inclusion/exclusion criteria. We included primary research (cross-sectional, qualitative and longitudinal surveys) focusing on the relationship/associations between CM and mental ill health. Papers included for full review were published in English language only.

**Table 2:** Inclusion and exclusion criteria, and outcomes of interest.

Inclusion criteria	Exclusion criteria	Outcomes of interest
**Articles discussing mental health of children married or promised in marriage before 18 years**	Articles that did not conduct subgroup analysis for mental health in children married or promised in marriage or married before 18 years.	Mental health status of children who were married or promised in marriage
**Articles discussing mental health of men and women aged 20–24 years who were married before the age of 18**	Mental health analysis in adult marriages	Factors that caused distress in child marriage
**Articles presenting results for children married before reaching the age of 18 separately if they were part of a larger sample**		Resulting mental health impacts of child marriage. These included: depression, psychological distress, anxiety, substance misuse and suicidality
**Articles comparing mental health in child marriage with mental health in adult marriages and with other forms of child abuse**		Other markers of wellbeing were life satisfaction, marital adjustment and satisfaction and self-esteem.
**Any country or setting**		
**Articles discussing distress related to terms outlined in section 3.2, in the context of marriage before the age of 18.**		
**Published in English**		

**Fig 1:**
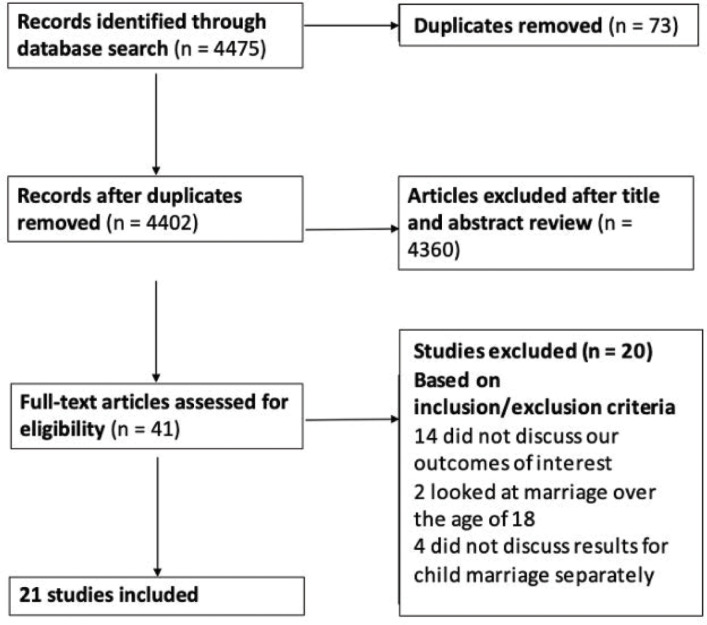
PRISMA flow diagram of studies included in the review.

**Fig 2:**
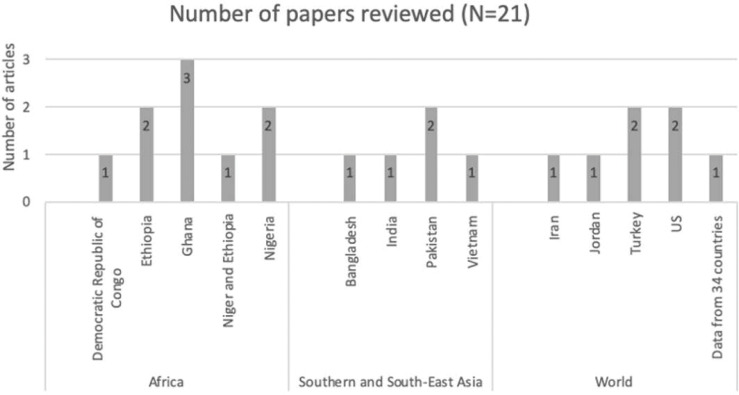
Countries examined in the articles included in our review.

Articles were screened using the Preferred Reporting Items for Systematic Review and Meta-Analysis (PRISMA) method [43] which is summarised in Fig 1. Titles and abstracts of the retrieved articles were reviewed against the inclusion criteria and duplicates removed. All titles and abstracts identified were screened independently by SAO and MJ. Disparities were resolved through discussion between SAO, MJ and RAB. Full texts of the selected articles were reviewed to confirm eligibility and the selected studies included in the review (Fig 2).

### 2.4. Quality assessment of the data

Quality assessments were completed by SAO and MJ using Joanna Briggs Institute Critical Appraisal Tools [44] using checklists for Qualitative, Analytical Cross-Sectional and Cohort Studies. The checklists had 10, 8 and 11 questions respectively assessing validity and reliability of studies. We adapted O’Donovan and colleagues [45] scoring system of a “traffic light” method to indicate where studies met criteria. Studies were scored as high or medium quality. No studies received a “low quality” score. See supplementary data tables for details of the quality assessment.

### 2.5. Summarising and reporting results

A data extraction table (see S1 Table) collected descriptive information including; author(s), year of publication, study population, sample size, research aims, study context, study design, methodology. For quantitative studies, additional outcome data including instrument type, descriptive and inferential statistics were extracted. For qualitative studies thematic categories were extracted.

### 2.6 Data synthesis

Given the heterogeneous nature of studies, we used a narrative synthesis approach [46]. This involved developing textual descriptions of individual studies then identifying common themes across studies. Qualitative and quantitative papers were assessed separately, and common themes identified. The search results were managed using Mendeley desktop software (Mendeley)

## 3. Results

### 3.1 Characteristics of studies

21 articles were included in our review, representing a range of countries, but concentrated in the African region (n = 9). Quantitative and qualitative papers are discussed under shared thematic headings but not compared to each other.

### 3.2 Mental health consequences of child marriages

**Fig 3:**
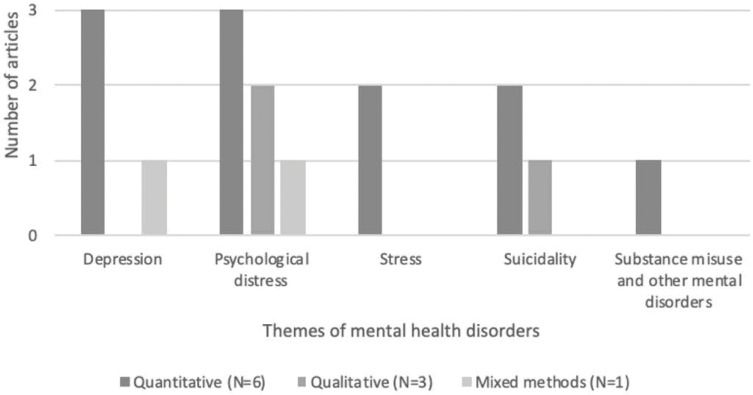
Thematic areas describing mental health disorders and factors identified in our review.

Our synthesis identified depression as the most common mental health consequence of CM (see Fig 3). Other issues include psychological wellbeing, stress, suicidality and substance misuse and other mental disorders.

#### 3.2.1 Depression

Depression was the specific condition explored the most in our sample, described in 3 quantitative and 1 mixed methods study [47–50]. In the US [48] dysthymia (persistent depressive disorder) had the strongest association to CM in the general population with increased odds of occurrence (2.20, 95% CI: 1.67–2.91) among women married as children compared to those married as adults. In Iran [49] early marriage increased depression risk by 2.77 times (AOR: 2.77, 95%; CI: 1.75–4.57; P = 0.001), even after adjusting for substance abuse, unemployment, age, residence, other negative life events (NLEs). In Turkey, Soylu et al. [50] compared mental health outcomes among early married girls and sexually abused girls, a comparison anchored to arguments that CM is a form of child abuse. Though both groups suffered from depression, sexually abused girls had a higher prevalence of Major Depressive Disorder (MDD) and Post-Traumatic Stress Disorder (PTSD). Those married early had higher rates of adjustment disorder.

#### 3.2.2 Psychological distress

Papers in this sample defined Psychological distress as a composite measure, often combining symptoms across a range of common mental health disorders, such as depression, anxiety and PTSD. Two quantitative and three qualitative studies found associations with CM and psychological distress [47, 50–53]. Two qualitative studies highlighted nuances around emotional distress. In Jordan [53] a study of 15 women aged 15–37 highlighted that early marriage and pregnancy contributed to distress associated with loss of education, self-confidence, decision-making power, childhood, freedom of mobility and peer social networks. In Ghana [52], girls who perceived their marriage as “early” reported increased distress linked to low income, pregnancy and childbirth and parenting challenges.

However, both studies also noted alleviation of some form in psychological distress in some cases, linked to increased access to financial and practical support (i.e childcare support) [53] or increased social status among girls whose marriage was framed as ‘timely’ instead of ‘early’ [52].

In Turkey, Soylu et al [50] aimed to compare sexually abused young girls (N = 72) referred for forensic evaluation and girls married early (N = 63), in terms of a number of variables such as mental health disorder rates, severity of mental health symptoms and sociodemographic characteristics. The participants had to complete a psychiatric evaluation in the form of an interview, and then a survey. The study demonstrated that while early marriage has extreme mental, social and physical outcomes, victims of sexual abuse showed higher rates of psychiatric disorders and more severe psychiatric symptoms.

In Ethiopia and Niger, psychological well-being (measured using the Psychological General Well-being Index) was negatively associated with marriage before 15 years [47] with well-being improving as age of marriage increased in Niger (β = − 7.41, SE: 2.26 at age 12 or earlier; β = −3.08, SE: 1.90 at age 17). Sub-domains of well-being were used to make a composite score, based on symptoms related to positive well-being, depression, anxiety, vitality and general health.

#### 3.2.3 Stress

Two cross-sectional studies examined the associations between CM and stress. In Ghana de Groot et al. [51] found a complicated relationship between stress and CM, that appeared to differ according to age. In a sample of women married before the age of 18, while there was a negative association between stress and CM among a sample of women aged 20–29 (Coefficient  =  − 1.18; CI -1.84 –-0.51), this not observed when looking at stress and CM for women aged 20–24. While the authors do not report precise regression coefficients for this age group, their analysis showed very wide confidence intervals, which perhaps signals the need for further investigation. In the US [48] those who married as children were more likely (24%) to report three or more stressful life events in the previous 12 months, compared to those married as adults (21%, p <0.001).

#### 3.2.4 Suicidality

Suicidal thoughts and attempts were identified in girls promised in marriage, girls who had received marriage requests and those in early marriages in two quantitative [50, 54] and one qualitative [55] study.

Gage’s [54] cross-sectional analysis identified that Ethiopian girls were twice as likely to report suicidal thoughts when promised in marriage or had received marriage requests (OR = 2.35; p < 0.01 and OR = 2.29; p < 0.01, respectively) compared to girls without marriage prospects. The odds of suicide attempts were also two times as high among girls who had received marriage requests (OR = 2.48; p <0.05). Geberesilase’s study [55] of girls married between 9–13 years old living with obstetric fistula noted suicidal thoughts and attempts alongside reports of isolation. Additionally, Soylu et al [50] reported that more girls who had experienced sexual abuse developed suicidal thoughts (79.2%) in comparison to girls who has experienced child marriage (34.9%).

#### 3.2.5 Substance misuse and other mental disorders and symptoms

One quantitative [48] and two qualitative studies [55, 56] explored the association between CM and substance misuse.

Le Strat et al., [48] reported that women married as children presented higher risk for, and significantly more mental disorders than those married as adults (35.50% vs 27.65%), with nicotine dependence and phobias as most common. Women married as children were also significantly more likely to be treated for any mental disorder in their lifetime (OR = 1.28 [95%CI: 1.09–1.52]), with anti-social personality disorder as the most common disorder, being three times as likely to be diagnosed with the condition (OR: 2.98 [95% CI: 2.03–4.37]) compared to those married as adults [48].

In their qualitative study, Gebresilase [55] reported that survivors of obstetric fistulas experienced feelings of powerlessness, isolation and avoidance. In another paper, Callaghan et al, [56] found that women who experienced CM reported feeling a loss of identity.

### 3.3 Key themes pertaining to drivers of emotional distress in child marriages

**Fig 4:**
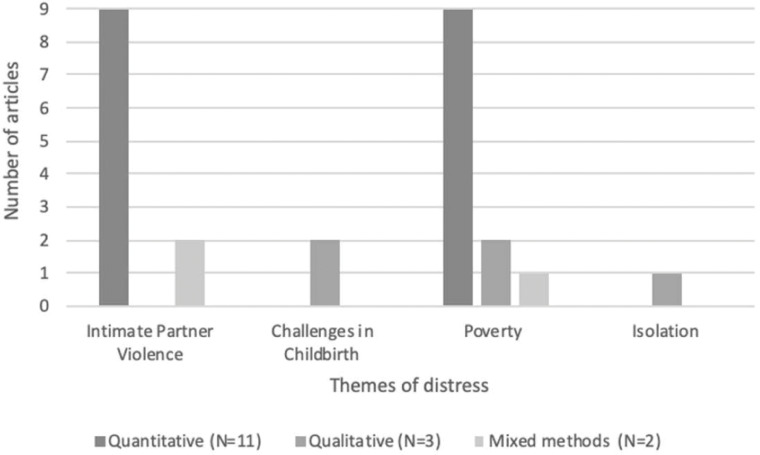
Thematic areas driving distress identified in review.

In our sample of papers, four socio-relational and socio-structural factors were mentioned in relation to emotional distress in CM contexts (see Fig 4): Intimate partner violence, poverty, challenges in childbirth and isolation.

#### 3.3.1 Intimate partner violence

IPV was discussed as a distressing factor in 12 studies, using varied definitions of the phenomenon, summarised in Table 3.

**Table 3:** Intimate partner violence reported in included studies.

Definitions of IPV used	Papers in Sample
**Collapsed all forms of IPV into one category**	Hong Le et al., [57] 2014; Wahi et al., [58] 2019, (N = 2)
**Physical IPV alone**	Yount et al., [59] (N = 1)
**Physical/sexual IPV**	Kidman, 2017 [60]; Raj et al., 2010, [61](N = 2)
**Physical/emotional IPV**	Nasrullah et al., 2014 [62](N = 1)
**WHO definition of IPV (physical violence, sexual violence, emotional violence and controlling behaviours)**	John et al., 2019 [47]; Landis et al., 2018 [63]; Sezgin & Punamaki, 2020 [64]; Tenkorang, 2019 [65]; Wusu, 2014 [66] (N = 6)

A cross-sectional study in Vietnam by Hong Le et al. [57] found that even when adjusting for other risk factors, early marriage was significantly associated with an increased risk of experiencing all forms of IPV [57]. Girls married before the age of 18 faced nearly twice the risk (AOR = 1.90; 95% CI, 1.12–3.21) of lifetime exposure to IPV compared with those married after 18. As one of two studies to consider men in our sample, they found no association between early marriage and the experience of IPV for young men [57].

In an American qualitative study, 18 of 21 participants who married early reported that their husbands had been either emotionally, physically or sexually abusive. Seven participants reported experiencing of all three forms of violence [58].

Five quantitative studies explored physical violence. In Pakistan, analysis of Demographic Health Survey (DHS) data indicated that physical violence was more common among women married as children than those married as adults (27.9% vs. 16.0%; p < 0.05) [62]. They also found that women married as children were two times as likely to experience either physical violence (OR = 2.03; 95% CI, 1.42–2.90) or physical and emotional violence (OR = 1.83; 95% CI, 1.33–2.50) and severe physical violence (7.3% vs. 3.1%; p < 0.05) (OR = 2.48; 95% CI, 1.23–4.98) [62]. Even after adjusting for a husband’s and girls’ sociodemographic characteristics (such as higher educated and >10 years older than their wives), CM was significantly associated with an increased likelihood of physical violence (AOR = 2.44; 95% CI, 1.58–3.76), and severe physical violence (AOR = 2.57; 95% CI, 1.12–5.87) [62]. Similar findings were noted in Ghana [65] (AOR = 1.86), however, risks was reduced by the access to family planning and economic decision-making (AOR = 1.77), education and employment (AOR = 1.56)

Yount et al. [59] worked in certain villages in Bangladesh and found where prevalence of very early CM (before age 15) was the lowest (<15%), incidence of physical IPV was 38.9%. As CM prevalence increased in villages, so did experiences of IPV, with incident rate of 44.1% in villages with moderate prevalence of very early CM (15%–25%), and 51.8% in villages with prevalence of very early CM over 25%. Authors also found that marrying in adulthood was protective against physical IPV (estimate = −0.29, SE = 0.11, p <0.01, OR = 0.75), but only in low prevalence villages [59]. Wusu [66] found a similar protective effect of marrying later in Nigeria, noting a negative association (OR = 0.94, p = <0.05) between age at first marriage and physical violence suggests older age of first marriage lowers risk of experiencing physical violence. In the US [64], the association between early marriage and women’s poor mental health outcomes showed that intimate partner violence was significantly associated to early marriage (*β* = −0.78, CR = 2.30, p < .05). No independent association between partner violence and poor mental health outcomes was found to be statistically significant.

#### 3.3.2 Sexual violence

In a study using DHS data of 39,877 women aged 20–24, from 34 low and middle-income countries across 7 regions (the Americas, East Asia & Pacific, Europe and Central Asia, Middle East & North Africa, South Asia, Eastern and Southern Africa and West and Central Africa), CM was a predictor of past year physical, sexual and combined IPV [60]. After adjusting for socio-demographic factors, CM increased the odds of physical and/or sexual IPV across age groups (AOR = 1.41 [95% CI 1.30–1.52] for marriage aged < 15; AOR = 1.42 (1.35–1.50) for marriage at 15–17). Risk for violence also varied across age of marriage. Marriage before 15 was associated with physical and sexual IPV in 9 countries, while those married between 15–17 faced increased risk in 19 countries. Heterogeneity between country results particularly in sub-Saharan Africa, reflects the varied explanations for CM and related risks for IPV, such as religious preference or economic necessity [60].

In the Democratic Republic of Congo (DRC), CM was not a protective factor against sexual violence even after controlling for girls’ ages and level of formal education [63]. The rate of forced sex in the previous 12 months was three times as high among married girls (36.4%) as compared to unmarried girls (12.8%), and highly significant (p<0.001) [63].

Distressing emotional violence was shown to be more prevalent among women who married as children compared to those married as adults in three quantitative studies [62, 63, 65]. Types of violence included threats, humiliation or insults by husbands [62, 63, 65], aggressiveness and neglect [63].

82.4% of girls who experienced CM in the DRC reported experiences of emotional violence compared to 62.4% of unmarried girls [63]. In Ghana, there were increased odds (AOR = 2.5) of females in CM experiencing emotional violence than those married as adults, although Tenkorang [65] reported that this reduced slightly after accounting for the endorsement of patriarchal gender norms, socio-economic characteristics, and indicators of autonomy (AOR = 1.98). In Pakistan, married girls were also found to have increased odds of experiencing emotional violence (OR = 1.70; 95% CI, 1.22–2.37).

Wusu’s [66] Nigerian study identified a range of intersectional factors linked to experience of emotional IPV among women married young in the country (mean age of marriage of 15.8 years). Significant predictors of experiences of emotional violence included age at first marriage, region (north vs. south of country), number of children, partner’s level of education and partner’s alcohol consumption.

### 3.4 Controlling behaviour and reduced agency

CM was linked to experiences of distress related to controlling behaviour in two quantitative and three qualitative studies. Using cross-sectional survey data, in Pakistan Nasrullah et al. [62] found women married as children reported experiencing more controlling behaviour vs. those married as adults (36.4% vs. 27.5%; p <0.05). They also had 52% increased odds of being controlled by their husbands (OR = 1.52; 95% CI, 1.12–2.08).

In Ghana de Groot et al. [51] women married early were less likely to believe their life was determined by their own actions (OR = 0.42; CI 0.25–0.72 for 20–24 and OR = 0.54; CI 0.39–0.75 for women 20 to 29). A second Ghanaian a cross-sectional survey found that women married as children reported lower autonomy related to sexual (n = −.35 vs n = .04; p <0.00) and family planning decision-making (n = −.33 vs n = .05; p <0.00) than those who were not [65].

Three qualitative studies explored controlling behaviour in CM. In Nigeria, women married as children highlighted a prevailing sense of being watched by their spouse, their spouse’s family and the community, and having restrictions on their movement and behaviour [65]. Wahi et al., [58] also reported controlling behaviours in CM in the USA, where eleven out of 21 participants described having restricted access to household finances or being forced to surrender their earnings. In their study in Ghana, Tenkorang [65] noted that women reported a similar sense of limited autonomy in their homes, with husbands holding all decision making power about their lives, Participants from the Nigerian study also discussed agency being limited outside the home, where they were watched by the community who expected them to act like a married woman, and transition to married life without complaint [56].

### 3.5 Challenges in childbirth

Complicated pregnancies and death of children were also identified as distressing factors in the Nigerian and Ethiopian qualitative studies [55, 56]. Participants from both studies were noted to be living with obstetric fistulae following childbirth, with narratives reflecting feelings of anger, sadness, and shame particularly due to the loss of productivity and support from husbands, family members, relatives, classmates, community members in the face of lost pregnancies.

### 3.6 Poverty

**Fig 5:**
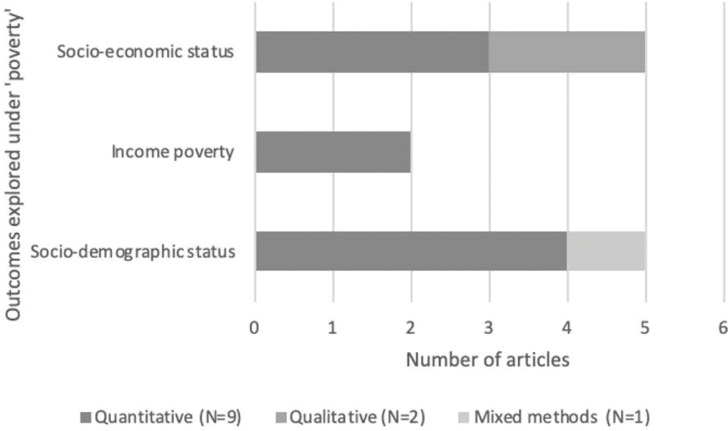
Outcomes explored related to poverty in papers included our review.

Socioeconomic status and poverty were explored in twelve papers (57%) (Fig 5). In five quantitative papers, socio-economic status was used as a confounding variable [47–49, 60, 61]. In Vietnam [57] higher rates of CM occurred in the lowest socio-economic status brackets. The poorest 20 percentile 15.7% of marriages were CM, compared to just 2.5% in the highest 20 percentile. In Pakistan [62] women married before age 18 had lower economic resources than those married above age 18.

Sociodemographic status was also explored, which included the analysis of variables such as education, smoking status, substance use, who the person lives with, paternal and maternal education, and the family’s monthly income [50].

Three papers treated poverty and low socio-economic status as a given consequence within CM. Yount et al. [59] defined poor households in terms of access to basic household characteristics, and found of households surveyed only 26.4% had electricity, and 1.8% had a flushing toilet. Though Sezgin and Punamaki [64] also found that poverty was an issue facing their respondents; 54% earned less than USD $280 a month, poverty was not explored in relation to mental health outcomes. De Groot and colleagues [51] found that 90% of their sample were living below the national poverty line.

Within two qualitative papers exploring distress [52, 56] discussion of poverty was present but links between the three phenomena were not explored.

### 3.7 Isolation

No quantitative studies looked at isolation. A qualitative Nigerian study highlighted that girls felt that their childhood had been ruptured following marriage, leading to isolation from family and friend networks, and the inability to return home [56].

## 4. Discussion

To our knowledge this is the first study to assess the literature pertaining to mental health consequences of CM globally. We identified numerous sources of emotional distress and mental health consequences linked to CM in peer reviewed literature. For many young people, this trajectory is likely to be worsened because of intersecting social challenges which carry their own negative impacts on mental health and wellbeing. Given that mental health problems in early- to mid-adolescence can have lasting impacts into adulthood [67, 68], rapid action in this area is needed.

Socio-structural and relational factors linked to distress did not occur in isolation; young and older women across studies reported intersecting structural factors at work in their lives. This clustering has potential negative implications for the severity of mental health outcomes, as studies from other contexts highlight that the experience of multiple adverse experiences in childhood is associated with more complicated mental health consequences in later life [69]. In addition to the major thematic areas presented in our findings, papers also suggested additional factors that may play a part in moderating mental health and child marriage associations, though their independent associations were not examined. These include loss of education, self-confidence, mobility, parenting challenges and other life events.

For qualitative studies that noted improvements in wellbeing linked to CM, this was linked to increased access to economic and social supports. In contexts of extreme poverty, marriage to older men carries economic benefits, with studies linking the continuation of the practice in many contexts to poverty and education [70]. Recent work by the Overseas Development Institute (ODI) suggests that CM is seen by parents as a way to extend social networks, optimise girls’ futures or improve the family’s economic situation [71]. However, given that our review also highlights that those married young still faced restrictions in autonomy in their marriages that contributed to emotional distress, is it possible that these positive gains could give way to more distressing relational dynamics over time.

Depression was the most explored mental health outcome in studies when taking into account composite measures. Girls have been reported to face greater risk of depressive symptoms and episodes due to increased exposure to stressful life events [72, 73]. For young women married early, depressive symptoms are associated to multiple social challenges including exposure to IPV. More broadly, depression in young people has been identified as a risk factor for substance misuse, social impairment and suicide [74]. Our findings also identified increased risk of suicidal ideations among this group of vulnerable women, which has been suggested as a route to punishing their families, given the high social stigma around death by suicide globally [75]. Given that recent work elsewhere highlights significantly higher levels of stigmatisation and shame in families where deaths by suicide had occurred after a two-year period [76], approaches that target entire families would be critical to alleviating this risk pathway.

We identified key gaps in the literature. At the time of the review there were no published studies exploring the mental health challenges linked to early marriage in humanitarian settings, or linked to sexual orientation, or disabilities. This is despite evidence highlighting the increasing rates of CM in such contexts [77–80]. Despite recent evidence suggesting that nearly 5% of young men aged 20–24 years report early marriage [81] we only identified two studies exploring the emotional and mental health consequences among boys married early. Furthermore, despite growing evidence exploring intergenerational impacts of mental health, including the ability for mental health conditions of mothers to impact on children through parenting and other pathways [82, 83] we were surprised that no studies explored intergenerational impacts of the mental health within households. Further research is needed to understand the complex power and gender inequities that are embedded within families and also contribute to poor mental health outcomes [84].

In light of our findings, we suggest that CM likely produces poor mental health outcomes not only due to the practice itself, but also linked to the impact of socio-structural factors at work in young people’s lives. However, the bi-directional relationship between these factors may be under appreciated. For example, while poverty was discussed in half of the studies it was often treated as a confounding variable rather than considering interactive effects. Given wide acknowledgement of the bi-directional relationship between mental health and poverty [26, 85] and its significant role in driving CM [15, 16] it must also be considered in the space of interventions for these populations alongside other key factors illuminated in this review, such as IPV, HIV, issues with pregnancy and childbirth, and agency.

To our knowledge, there are currently no intervention programmes which focus directly on the mental health needs of those married young. While we note the need for future studies to explore directionality in the associations discussed in this paper, existing literature does support speculation on the potential shape of mental health supports for this population. Table 4 sets out recommendations for mental health supports staggered across the staged model of disorder discussed in our conceptual framework. These recommendations centre recent calls by Burgess and others, for interventions that engage equally with psychological distress and wider social factors that produce or deepens distress [86], as they are two sides of the same coin.

**Table 4:** Potential solutions for targeting mental distress linked to child marriage.

Staging Classification	Symptoms/conditions	Suggested solution	Examples	References	Population
Stage 0–1 asymptomatic and non-specific mental distress	*Emotional symptoms*: Distress, loneliness, stress	School based mental health supports	Mental health literacy interventions	Rajaraman et al., [92]	For girls returning to school; for those facing the threat of child marriage
		Mental health promotion and prevention through social change	Eg: BALIKA programme with livelihoods, education and gender rights awareness training supports.	Amin et al., 2018 [87]	For adolescents in the community
	*Social drivers*: poverty, IPV, isolation	Community engagement and development interventions	Group Interventions to promote women’s maternal health (PLA groups)	Sondaal, 2018 [93]	Women married young in the community
Stage 2–4 Emphasis on Common Mental disorders	*Specific conditions*: Depression, suicide risk, substance misuse	Packages of care for common mental disorders, including those targeting suicide risk	Problem solving+;	Dawson et al., 2015 [94]	For women with clinical symptoms, delivered in various settings
			Community based therapies (schools)	Brown et al., 2019 [95]	For older adolescents returning to school
			Group based therapy combined with livelihood supports	Gumbonzvanda et al., 2021 [91]	For women married as children–community based-support
	*Social drivers*: poverty, IPV, reduced agency	Community engagement to promote social change	PLA groups for mental health improvements	Belaid et al., 2021 [96]	For women and wider community members (including male partners)

For example, for stages 0–1, we draw attention to existing interventions designed to reduce CM prevalence through action on social drivers such as poverty and isolation, as they also potentially contribute to mental health promotion. For example, the Bangladeshi Association for Life Skills, Income, and Knowledge for Adolescents (BALIKA) program which successfully reduced risk of CM [87] targets social drivers also linked to poor mental health that when addressed have improved mental health outcomes for young women living in adversity elsewhere [88, 89]. In working at stages 2–4, mental health interventions should not exclude spaces to address the wider contexts that drive or deepens distress. For example, we highlight the value of problem-solving interventions which have been used widely across low- and high-income settings [6].

Additional therapies which deploy a trauma informed approach would be key to supporting women married young. Given the embeddedness of these women within community life, collective and participatory interventions could be valuable. For example, Collective narrative therapy, which has been shown to be effective in community-based settings [90] (Burgess et al., 2021) could also resonate with these populations, through their ability to centre collective action while restoring traumatic histories.

We also suggest the value of exploring community-based interventions which work on the wider structural drivers of both poor mental health and CM. For example, Participatory, learning and action groups (PLA) could provide platforms for young women to engage with networks, despite restrictions on their agency, their more general focus on topics that may be more acceptable to male partners (i.e women’s health issues, livelihoods, etc). Recent work in Zimbabwe suggests that PLA style interventions that centre dialogue in can shift gendered relational dynamics that contribute to child marriage, such as local law and custom, while promoting emotional literacy and wellbeing [91]. Overall, these suggestions provide a useful starting point for policy actors and services to address the immediate need to provide mental health support for those currently in such marriages. Solutions should prioritise common mental disorders using community-led interventions and approaches to centre people’s voices, and the struggles of their lives that shape poor mental health, using low-cost interventions.

### 4.1 Limitations

Our review has several limitations. The cross-sectional nature of data and limited number of cohort studies limits our ability to understand causality. Most studies relied on self-report data which may have introduced social desirability bias, especially given the sensitive nature of data. This suggests that longitudinal methods that place ownership of narratives in young peoples’ hands (qualitative cohort studies) may yield valuable insights in the future. Second, though grey literature shaped our initial scoping review, it was not included in the full review, which may have overlooked smaller scale studies conducted by NGO’s and other public sector organisations. For example, recent evidence by UNICEF and ODI highlighted the issue of migration as a means of avoiding early marriage [71], which would likely establish additional mental health effects not captured in our analysis. We also noted difficulties linked to the comparison groups used within some studies, for example, CM as defined as forms of child trafficking [97] or sexual abuse and exploitation [41, 98]. Finally, future studies should explicitly explore non-English publications, as many countries where child marriage is prevalent may also include reports or publications in additional languages (such as French and Portuguese).

## 5. Conclusion

Depression, anxiety, suicidal thoughts and attempts, as well as emotional distress caused by poverty, IPV, isolation, challenges in childbirth, self-esteem and loss of autonomy were associated with CM in the literature. Further research will enable a more comprehensive understanding of the pathways to the development of mental health conditions related to CM. Studies should explore the needs of boys married early, and family driven pathways to mental health outcomes following CM. Global efforts to bring an end to this harmful practice will only be strengthened by parallel efforts to ensure psychological wellbeing for the millions of young people and communities affected by this practice.

## Supporting information

S1 Appendix (DOCX)

S1 Text Sample search–pubmed search engine. (DOCX)

S1 Table Study characteristics. (DOCX)

S2 Table Summary of key findings pertaining to drivers of emotional distress. (DOCX)

S3 Table Summary of key findings pertaining to mental health outcomes. (DOCX)

S1 Fig Summary of methodological quality of cross-sectional studies using Joanna Briggs Institute Critical Appraisal checklist. (EPS)

S2 Fig Summary of methodological quality of qualitative studies using Joanna Briggs Institute Critical Appraisal list. (EPS)

S3 Fig Summary of methodological quality of longitudinal studies using Joanna Briggs Institute Critical Appraisal list. (EPS)
