# Supplementary material for: Overlooked and unaddressed: A narrative review of mental health consequences of child marriages
Source: PLOS Glob Public Health. 2022 Jan 12;2(1):e0000131. doi: 10.1371/journal.pgph.0000131 (PMC10021205; doi:10.1371/journal.pgph.0000131)
Supplement: S1 Appendix — (DOCX) [file pgph.0000131.s001.docx]

**PubMed Literature search**

(“early marriage” OR “child marriage” OR “prepubescent female marriage” OR “child bride” OR “teenage marriage” OR “adolescent marriage” OR “child widowhood” OR “forced marriage” OR “marriage market” OR “maternal child marriage” OR “bush wives” OR “arranged marriage” OR “arranged union” OR “too early marriage” OR “bride kidnap”) AND (“mental health” OR suicide OR suicidal OR “suicidal thoughts” OR suicidality OR “self harm” OR “self-harm” OR depression OR anxiety OR “emotional distress” OR stress OR “maternal mental health” OR “perinatal mental health” OR “substance misuse” OR “substance abuse” OR PTSD OR “post-traumatic stress disorder”)
