# Supplementary material for: Overlooked and unaddressed: A narrative review of mental health consequences of child marriages
Source: PLOS Glob Public Health. 2022 Jan 12;2(1):e0000131. doi: 10.1371/journal.pgph.0000131 (PMC10021205; doi:10.1371/journal.pgph.0000131)
Supplement: S1 Table — (DOCX) [file pgph.0000131.s003.docx]

| **S1 Table: Study Characteristics** | | | | | | | |
| --- | --- | --- | --- | --- | --- | --- | --- |
| **Authors** | **Year** | **Location of Study** | **Aim** | **Design/Methodology** | **Population (n =)** | **Gender (n =)** | **Age range (years)** |
| Callaghan, Gambo and Fellin | 2015 | Nigeria | Report on experiences of women from northern Nigeria, who were married under the age of 16. | Qualitative study, using interviews and interpretive phenomenological analysis | Nigerian women (n = 6) from Sokoto state, who were married between 8 and 15 | Female = 6 | 25–35 years |
| de Groot, Kuunyem and Palermo, | 2018 | Ghana | To investigate whether child marriage is associated with lower levels of agency and social support, and higher levels of self-perceived stress. | Cross-sectional study using data from a baseline survey of a longitudinal impact evaluation. | Ever-married women (n = 1349) from 2497 households in the Northern and Upper East regions of Ghana | Female = 1349 | 20–29 years |
| Gage | 2013 | Ethiopia | To investigate the association between child marriage and suicidal ideation and suicide attempts in girls aged 10 to 17 years. | Cross-sectional study using data from a 2007 survey conducted in the Amhara region in Ethiopia. | Girls (n = 2,709) were interviewed in the Amhara region, Ethiopia | Female = 2709 | 10-17 years |
| Gebresilase | 2014 | Ethiopia | To report on women’s perspectives of their social relationships and health after surviving obstetric fistula. | Qualitative study using the theoretical framework of the ecological model and the Transactional model of stress and coping. | Survivors (n = 8) of obstetric fistula | Female = 8 | 18–24 years |
| John, Edmeades and Murithi | 2019 | Niger and Ethiopia | To investigate the association between child marriage and psychological well-being. | Mixed methods study. Quantitative data were from a multi-country study to estimate the economic costs of child marriage. Qualitative data from in-depth interviews and focus groups. | Ever-married women in Niger (n = 2764) and Ethiopia (n = 4149) and some parents who had daughters between age 8-17 years | Female = 6913 | 18–45 years |
| Landis et al | 2018 | DRC | To investigate the association between child marriage and level of participation in formal education, exposure to violence, and well-being. | Baseline survey from 14 communities in South Kivu in the Democratic Republic of the Congo. | Girls (n = 350), 87 of whom were married or living with a partner as if married | Female = 350 | 13-14 years |
| Le Strat, Dubertret and Le Foll | 2011 | USA | To investigate the prevalence of psychiatric disorders associated with child marriage rates of mental health treatment-seeking. | Cross-sectional study using data from the 2001–2002 National Epidemiologic Survey on Alcohol and Related Conditions | Women (n = 24,575) who had been or were presently married, with a known age of first marriage | Female = 24,575 | 18-65+ years |
| Nasrullah, Zakar and Zakar | 2014 | Pakistan | To investigate the relationship between child marriage and controlling behaviour and spousal violence in Pakistan. | Cross-sectional survey | Data from the Pakistan Demographic and Health Survey, 2012 - 2013, of currently married women who had participated in the domestic violence module (n = 589) | Female = 589 | 15 - 24 years |
| Raj et al. | 2010 | India | To investigate whether child marriage is associated with an increased risk of physical or sexual marital violence in India. | Cross-sectional survey | Married women n = 10,514) who participated in the marital violence survey module in the 2005-2006 India National Family Health Survey 3. | Female = 10 514 | 20–24 years |
| Segzin and Punamäki | 2020 | Turkey | To retrospectively examine the impacts of early marriage and adolescent pregnancy on self-reported mental and physical health; To analyse if and how partner violence moderates the relationships between these factors and mental health problems | Cross-sectional survey | Married women (n = 1569) | Female =1569 | 16-72 years |
| Shaud and Asad | 2018 | Pakistan | To investigate the differences in marital adjustment, convergent communication patterns, and psychological distress in early and late marriages. | Cross-sectional survey | Women (n = 100); 50 who got married either at 18 or before 18 years of age and 50 women who first got married either at 30 or above 30 years of age | Female = 100 | Mean early marriage = 25.84  Mean age late marriage = 43.04 |
| Soylu, Ayaz and  Yüksel | 2014 | Turkey | To investigate the difference in psychiatric outcomes between girls who had been forced into early marriage, and girls who had been sexually abused. | Cross-sectional survey | Early-married girls (n = 63), who had been sent to the Child Psychiatry Outpatient Department of Gaziantep Children’s Hospital, Turkey for treatment and a judicial report; and 72 sexually abused girls, who had been sent to the same hospital | Female = 63 |  |
| Tenkorang | 2019 | Ghana | To examine the relationship between child marriage and intimate partner violence (IPV) | Data from a nationally representative cross-sectional survey in 2017, as one part of a bigger study on domestic violence. | Ever-married Ghanaian women (n = 2289) | Female = 2289 | Mean age = 38 years |
| Al-Kloub et al. | 2019 | Jordan | To explore the experiences of early marriage and motherhood of women in Jordan. | Qualitative study | Jordanian women (n = 15) aged who had given birth before 19 years of age | Female = 15 | 15-37 years |
| Baba et al. | 2020 | Ghana | To explore the experiences of well- being and challenges of married girls in Ghana. | Qualitative study | Married girls (n = 21) | Female = 21 | 12-19 years |
| Fakhari et al. | 2020 | Iran | To investigate the relationship between early marriage and negative life events and depression in adolescents. | Cross-sectional survey | 530 Respondents (n = 530), consisting of 26% adolescents (aged 13–19), 45% young adults (aged 20–33), and 28% adults (aged 33–40) | Female = 300  Male = 230 | 13-40 years |
| Hong Le et al. | 2014 | Vietnam | To investigate the prevalence and associations between early marriage and IPV among adolescents and young adults in Vietnam | Secondary analysis of data from the 2009-2010 National Survey Assessment of Vietnamese Youth–Round II (SAVY-II) | Participants (n = 10,044), 6,508 aged 14-19, and 1,468 ever-married 20-25-year-old young adults | Female = 10,044 | 14-25 years |
| Wusu, O. | 2014 | Nigeria | To investigate the prevalence of different forms of IPV against married girls, the predictors of IPV and the association between forms of IPV and indicators of sexual and reproductive health. | Cross-sectional study using data from the 2008 Demographic and Health Survey in Nigeria | Married girls (n = 4574) | Female = 4574 | 15-24 years |
| Yount et al. | 2016 | Bangladesh | To investigate the relationship between child marriage and IPV and how village-level prevalence of very early child marriage influences a woman’s risk of experiencing IPV. | Longitudinal study | Recently (within 4-12 years) married women (n = 3,355) | Female = 3,355 | 16-37 years |
| Kidman, R. | 2017 | 34 countries | To investigate whether women who married as children are at increased risk of past-year physical and/or sexual IPV, compared with adult-married women. | Cross-sectional survey | 34 demographic and health surveys (DHS) were used for this study. The prevalence of child marriage was calculated for women aged 20–24 in the domestic violence sample of the DHS (n = 39,877) | Female = 39,877 | 20-24 years |
| Wahi et al. | 2019 | USA | To report on the experiences of married American children. | Qualitative study using an online questionnaire and phone interviews. | Participants (n = 21) married between 13-17. | Female = 20  Male = 1 | 26-74 years |
