# Supplementary material for: Overlooked and unaddressed: A narrative review of mental health consequences of child marriages
Source: PLOS Glob Public Health. 2022 Jan 12;2(1):e0000131. doi: 10.1371/journal.pgph.0000131 (PMC10021205; doi:10.1371/journal.pgph.0000131)
Supplement: S2 Table — (DOCX) [file pgph.0000131.s004.docx]

| S2 Table: Summary of key findings pertaining to drivers of Emotional distress | | | |
| --- | --- | --- | --- |
| Drivers of mental health vulnerability | **Related studies** | **Outcomes** | **Key findings** |
| Intimate partner violence | Hong Le et al., 2014 | Intimate partner violence | - Early marriage was significantly associated with an increased risk of experiencing IPV among young women. - Marriage before age 18 gave girls and young women nearly twice (AOR=1.90; 95% CI, 1.12–3.21) the risk of lifetime exposure to IPV, compared with those who were aged at least 18 when married. - In young men, there was no association found between adolescent marriage and experiencing IPV. - Married girls and young women were more likely to report being verbally, physically, and/or sexually abused by an intimate partner than young married men (24.8% vs. 19.2% respectively). - More young men (25.1%) than young women (18.9%) perceived their partners as behaving in controlling ways and prohibiting them from doing things that they wanted to do. |
|  | Wahi et al., 2019 | The lived experience of child marriage | - Most participants (n = 18) reported physical, sexual, financial, or emotional abuse during their marriage as well as unwanted and/or unplanned pregnancies. - Fewer than half (n = 10) participants reported being allowed by their spouses to use contraception if they wanted. |
|  | Yount et al., 2016 | Intimate partner violence | - Experiences of physical IPV and child marriage were common among recently married women. Almost one-half (44.5 %) of the women reported exposure to physical IPV between the baseline survey and follow-up. - In villages where very early child marriage was lowest (<15%), the incidence of physical IPV was 38.9%. The incidence rate was higher (44.1%) in villages with a moderate prevalence of very early child marriage (15%–25%). - The incidence was highest (51.8%) in villages with a high prevalence of very early child marriage (>25 %). - Marrying in adulthood was found to be slightly protective against experiencing physical IPV (estimate = −0.29, SE = 0.11, p < .01, OR = 0.75). |
|  | Raj et al., 2010 | Intimate partner violence (marital violence) | - Study found that 43% of girls who got married as adolescents reported marital violence compared to 24% who married as adults (AOR=1.77; 95% CI, 1.61–1.95) and were more likely to report violence in the past 12 months (AOR=1.51; 95% CI,1.36–1.67). - Girls married in adolescence were twice as likely to report severe physical marital violence (16%) compared to those who married as adults (AOR=2.04; 95% CI, 1.75–2.38) and in the last 12 months (AOR=1.82; 95% CI, 1.54–2.16) |
|  | Landis, 2018 | Sexual, physical and emotional violence | - The study found a significant association between child marriage and a higher occurrence of violence. - Marriage was associated with an increased likelihood of experiencing sexual violence. It was nearly three times as high (OR: 2.79) for married as compared to non-married girls. - When adjusted for age and girls’ level of participation in formal education, being married was associated with more than a three-fold (OR: 3.23) increased risk of experiencing sexual violence (p<0.001). |
|  | Tenkorang, 2019 | Intimate partner violence (physical, sexual and emotional) | - Most respondents reported emotional violence (58%), and a large number reported physical and sexual violence (39.8% and 34.6% respectively). - Women who married as children were more likely to experience physical violence (AOR = 1.86), sexual violence (AOR = 1.33) and emotional violence (AOR = 2.5) than those who married as adults. |
|  | Kidman, 2017 | Intimate partner violence | - Past year physical and/or sexual IPV was higher among women who married as children (29%) compared to those who got married as adults (20%). - Being married as a child compared to as an adult increased the odds of physical and/or sexual IPV: adjusted odds ratio (AOR = 1.41 [95% CI 1.30–1.52] for marriage aged < 15; AOR = 1.42 (1.35–1.50) for marriage at 15–17 (after controlling for socio-demographic factors). - There was heterogeneity between countries: marriage before age 15 was associated with a combined measure of past year physical and/or sexual IPV in nine countries; women married between 15 and 17 were at increased risk of physical and/or sexual IPV in 19 countries. - Heterogeneity between countries was most evident in sub-Saharan Africa. |
|  | Wusu, 2014 | Intimate partner violence | - The study found that the significant predictors of IPV included age, region, age at first marriage, education, wealth status, number of living children, spouse’s education, jealousy, and alcohol consumption. - The study found there was a negative association (OR = 0.938, p = 0.05) between age at first marriage and physical violence experienced by respondents, suggesting that the older the age at marriage, the less likely such women would have ever experienced physical violence. |
|  | Nasrullah, Zakar, and Zakar, 2014 | Intimate partner violence (including marital control, emotional and physical violence). | - Women who were married as children reported experiencing more controlling behaviour than those married as adults (36.4% vs. 27.5%; p < .05) - Physical violence (27.9% vs. 16.0%; p < .05), including severe physical violence (7.3% vs. 3.1%; p < .05) was more prevalent among women married as children compared with those married as adults. - Girls married as children were twice as likely (OR = 2.48; 95% CI, 1.232-4.977) to have ever experienced severe physical violence compared with those married as adults. - Women married as children compared with women married as adults were more likely to have experienced controlling behaviours by their husbands (OR = 1.52; 95% CI, 1.117-2.082). |
|  | John, Edmeades, and Murithi, 2019 | Psychological well-being  Intimate Partner Violence (IPV) | - IPV was strongly negatively associated with psychological well-being in child marriages - Early married girls in Ethiopia experienced physical violence from husbands especially if they refused to comply to their sexual demands |
|  | Sezgin and Punamäki | Intimate partner violence | - The study found that early marriage and adolescent pregnancy were not associated with women’s mental health problems, through the pathway of high levels of intimate partner violence. |
| Challenges in childbirth |  |  |  |
|  | Callaghan, Gambo, and Fellin 2015 | The women’s perspective of their experience of early marriage | - Loss of their own identity and self-esteem, especially when they experienced complicated childbirth resulting in fistula or death of a child |
|  | Gebresilase, 2014 | The experience of living with obstetric fistula | - Obstetric fistula was found to have significant physical, psychosocial, and emotional consequences including associations with feelings of anger, sadness, shame over the loss of a child; loss of ability to work; loss of acceptance and support by their husbands, family members, relatives, classmates, community members, and other passengers when using public transport. |
| Poverty | John et al., 2019; Kidman 2017; Raj et al., 2010; Fakhari, 2020; Le Strat et al 2011 | Socio-demographic status | - Used as a confounding variable |
|  | Hong Le et al. (2014) | Socio-economic status | - A higher percentage of child marriages happened within the lowest socio-economic status. The poorest 20% had 15.7% of child marriages, compared to 2.5% in the highest 20%). |
|  | Nasrullah et al. (2014) | Socio-economic status | - Women married before age 18 were poorer and less educated than women who married above age 18. |
|  | Callaghan, Gambo, and Fellin, 2015 | Socio-economic status | - All participants were from families suffering from poverty. |
|  | de Groot et al., 2018 | Income poverty | - 90% of their sample of 594 women was living below the national poverty line. |
|  | Baba, Salifu Yendork, and Atindanbila, 2020 | Socio-economic status | - Most respondents had a low socio-economic status |
|  | Sezgin and Punamaki, 2020 | Income poverty | - High rates of poverty in respondents - 54% earned less than USD $280 a month. |
|  | Yount et al., 2016 | Socio-economic status | - Most respondents were from poorer households with low educational status.   26.4% had electricity in their homes   - 1.8% had a flushing toilet within the home |
|  |  |  |  |
| Isolation | Callaghan, Gambo, and Fellin, 2015 | The women’s perspective of their experience of early marriage | The participants explained their experiences as:   - Loss of contact with their natal family abruptly rupturing their childhood; - Physical restrictions placed on them e.g. not being allowed to go out without their husbands; - Limited agency - prevailing sense of "being watched" and doing only what their husbands want them to do curtailing their freedom; - Loss of their own identity and self-esteem especially when they experienced complicated childbirth resulting in fistula or death of a child - Vulnerability when the marriage does not go well, and they are rejected by their husbands and his family - A failure to have a voice of their own - the men are in charge therefore women have to listen and obey despite being the ones who have to bear the burden of complications arising from early marriage |
