# Supplementary material for: Overlooked and unaddressed: A narrative review of mental health consequences of child marriages
Source: PLOS Glob Public Health. 2022 Jan 12;2(1):e0000131. doi: 10.1371/journal.pgph.0000131 (PMC10021205; doi:10.1371/journal.pgph.0000131)
Supplement: S3 Table — (DOCX) [file pgph.0000131.s005.docx]

| **S3 Table: Summary of key findings pertaining to mental health outcomes** | | | |
| --- | --- | --- | --- |
| **Mental health outcomes** | **Related studies** | **Study measures** | **Key findings** |
| Depression | Le Strat, Dubertret, and Le Foll, 2011 | Diagnostic Assessment and Mental Health Service Utilisation | - The strongest association between child marriage and mental disorders is dysthymia (persistent depressive disorder), with increased odds (2.20, 95% CI: 1.67–2.91) of occurrence among women married as children compared to women married as adults. |
|  | Soylu, Ayaz, and Yuksel, 2014 | Subscales of the Brief Symptom Inventory (BSI): somatisation, OCD, interpersonal sensitivity, depression and anxiety disorder | - At least one psychiatric disorder was determined in 44.4% of the early-married and 77.8% of the sexually abused cases (p < 0.001). - Major depressive disorder was seen in 33.3% of early-married cases and 56.9% of sexually abused cases (p = 0.006). - All of the subscale scores of the BSI were higher in the sexually abused adolescents than in the early-married cases (p < 0.001). - Early marriage was determined to have severe physical, social and mental outcomes but not as severe as sexual abuse in terms of psychiatric disorder rates and the psychiatric symptom severity it causes. |
|  | Fakhari, 2020 | Depression | - This study found a significant relationship between depression and early marriage (2.77; CI: 1.75–4.57) after adjusting for age, residence, substance abuse, alcohol abuse, unemployment and other negative life events. - Early marriage was the most statistically significant determinant that increased the odds of depression. - 46% of all respondents had symptoms of depression, (24% had mild, 14% had moderate and 8% had severe depression). |
|  | John, Edmeades, and Murithi, 2019 | Psychological well-being  Intimate Partner Violence (IPV) | - Very early marriage was negatively associated with psychological well-being. - In Niger very early marriage was associated with poor psychological well-being which improved with increasing age at first marriage |
| Psychological distress | Al-Kloub et al., 2019 | The lived experience of marriage and motherhood in young women | - Participants reported feelings of a sense of loss, e.g. education, which undermined their self-confidence and decision-making power, and denied them the opportunity to become economically independent. - Participants reported a loss of childhood and youth, personal freedom for mobility and peer social networks. - Some positive experiences were expressed, with financial and practical support from family members regarding childcare. |
|  | Baba, Salifu Yendork, and Atindanbila, 2020 | Married girls’ perception of the timing of their marriage  Subjective experiences of well-being and challenges as young wives and young mothers | - The study suggests that girls who perceived their marriage as “early” reported negative emotions whereas those who perceived their marriage as “timely” reported positive emotions. - Married girls’ positive experiences were characterised by childbearing, higher social status, received social support and satisfaction of needs in their marriage. - Challenges identified included poor socio-economic status, inadequate parenting skills, pregnancy and childbirth related distresses. |
|  | John, Edmeades, and Murithi, 2019 | Psychological well-being | - Psychological well-being was negatively associated with very early marriage (<15 years). - Well-being improved as age of marriage increased in Niger (β = − 7.41, SE: 2.26 at age 12 or earlier; β = −3.08, SE: 1.90 at age 17.23). - Sub-domains of well-being (depression, anxiety, positive well-being, vitality and general health) were negatively associated with very early marriage. |
|  | de Groot et al., 2018 | Health, fertility, contraception, child mortality, social support, stress and agency outcomes | - Child marriage was associated with increased odds of poorer health (measured by difficulties in daily activities) (OR = 2.08; CI 1.28–3.38 among women 20–24 years and OR = 1.58; CI 1.19–2.12 among women 20– 29 years); - Also associated with increased odds of child mortality among first-born children (OR = 2.03; CI 1.09–3.77 among women 20–24 years); - Lower odds of believing that one’s life is determined by one’s own actions (OR = 0.42; CI 0.25–0.72 among women 20–24 years and OR = 0.54; CI 0.39–0.75 among women 20–29 years). - Lower levels of reported stress (regression coefficient = − 1.18; CI -1.84–-0.51 among women 20–29 years). |
|  | Soylu, Ayaz, and Yuksel, 2014 | Post-traumatic stress disorder (PTSD) | - PTSD or ASD was seen in 11.1% of early-married cases and in 54.2% of the sexually abused victims (p < 0.001). |
|  | Shaud and Asad, 2018 | Marital adjustment, convergent  communication patterns, and psychological distress | - Women with late marriages had a higher marital adjustment, while women with early marriages showed higher psychological distress - Early-married women were more likely to exhibit convergent communication patterns and interpersonal deference. - Women who got married early had greater difficulty adjusting to their marital life and experienced more psychological distress than those who married later. |
| Stress | Le Strat, Dubertret, and Le Foll 2011 | Diagnostic Assessment and Mental Health Service Utilisation | - Women who married as children were more likely to report >/=1 stressful life event within the past 12 months, compared with adult-married women. |
|  | de Groot et al. 2018 | Stress | - There was a greater association between early marriage and stress in women aged 20-24 compared to the age 20-29 age group. - Lower levels of reported stress (regression coefficient = − 1.18; CI -1.84–-0.51) among women aged 20–29 years. |
| Suicidality | Gage, 2013 | Suicidal thoughts and suicide attempts | - The odds of suicidal ideation were 1.81 (p <.05) times as high among ever-married girls and twice as high among girls promised in marriage (odds ratio [OR]= 2.35; p <.01) and those with marriage requests (OR = 2.29; p < .01) as compared to those reporting no marriage requests. - Suicide attempts were higher (OR = 2.48; p <- .05) among girls with marriage requests as compared to those never in the marriage process. |
|  | Gebresilase, 2014 | Suicidal thoughts and attempts | - Some participants (n = 6) had experienced suicidal thoughts and one had attempted suicide. - They study reported that following stressful events some participants used an emotion-focused coping strategy, such as isolating themselves, suicidal thoughts and attempts, avoidance or positive subjective interpretation. |
|  | Soylu, Ayaz, and Yuksel, 2014 | Suicidal thoughts and attempts | - Study found that more girls (79.2% or n = 57) who had experienced sexual abuse developed suicidal thoughts than girls who had experienced early marriage (to 34.9% or n = 22). - More girls who had experienced sexual abuse had suicide attempts (37.5% or n = 27) than early married girls (26.9% or n = 17) |
| Substance misuse and other mental disorders | Le Strat, Dubertret, and Le Foll, 2011 | Diagnostic Assessment and Mental Health Service Utilisation | - Women who were married as children had significantly more mental disorders than women who got married as adults (35.50% vs 27.65%). - The risk of women who were married as children developing nicotine dependence and phobias increased by 2.02 and 1.35, compared to women who got married as adults. - Women who married as children were significantly more likely to receive lifetime treatment for any mental disorder ((OR = 1.28 [95%CI: 1.09–1.52]) compared to women who got married as adults. - The odds of developing anti-social personality disorder nearly three times (OR: 2.98 [95% CI: 2.03– 4.37]) greater in women married as child compared to women who got married as adults. |
